# Supplementary material for: First Report of Antimicrobial Susceptibility and Virulence Gene Characterization Associated with Staphylococcus aureus Carriage in Healthy Camels from Tunisia
Source: Animals (Basel). 2021 Sep 21;11(9):2754. doi: 10.3390/ani11092754 (PMC8468875; doi:10.3390/ani11092754)
Supplement: Supplementary file 1 [file animals-11-02754-s001.zip › animals-1301424-supplementary.pdf]

Supplementary Table: Primers, amplicon size, and amplification conditions of staphylococcal virulence genes

Virulence genes	Primers		Sequence (5’–3’)	Size	PCR conditions
Enterotoxin genes	sea	SEA-1<br>SEA-2	GAAAAAAGTCTGAATTGCAGGGAACA<br>CAAATAAATCGTAATTAACCGAAGGTTC	560	95 ◦C × 5 min, 35 × (95 ◦C × 30 s, 55 ◦C × 30 s, 72 ◦C × 1 min) 72 ◦C × 10 min, 4 ◦C
	seb	SEB-1<br>SEB-2	ATTCTATTAAGGACACTAAGTTAGGGA<br>ATCCCGTTTCATAAGGCGAGT	404	
	Sec	mpSEC-1<br>mpSEC-2	GTAAAGTTACAGGTGGCAAAACTTG<br>CATATCATACCAAAAAGTATTGCCGT	297	
	sed	SED-1<br>SED-2	GAATTAAGTAGTACCGCGCTAAATAATATG<br>GCTGTATTTTTCCTCCGAGAGT	492	
	see	SEE-1<br>SEE-2	CAAAGAAATGCTTTAAGCAATCTTAGGC<br>CACCTTACCGCCAAAGCTG	482	95 ◦C × 5 min, 35 × (95 ◦C × 1 min, 55 ◦C × 1 min, 72 ◦C × 1 min) 72 ◦C × 10 min, 4 ◦C
	seg	SEG-1<br>SEG-2	AATTATGTGAATGCTCAACCCGATC<br>AAACTTATATGGAACAAAAGGTACTAGTTC	642	95 ◦C × 5 min, 35 × (95 ◦C × 2 min, 55 ◦C × 2 min, 72 ◦C × 1 min) 72 ◦C × 10 min, 4 ◦C
	seh	SEH-1<br>SEH-2	CAATCACATCATATGCGAAAGCAG<br>CATCTACCCAAACATTAGCACC	376	
	sei	SEI-1<br>SEI-2	CTCAAGGTGATATTGGTGTAGG<br>AAAAAACTTACAGGCAGTCCATCTC	576	95 ◦C × 5 min, 35 × (95 ◦C × 45 s, 62 ◦C × 45 s, 72 ◦C × 1 min) 72 ◦C × 10 min, 4 ◦C
	sej	mpSEJ-1<br>mpSEJ-2	TAACCTCAGACATATATACTTCTTTAACG<br>AGTATCATAAAGTTGATTGTTTTCATGCAG	300	95 ◦C × 5 min, 30 × (95 ◦C × 1 min, 63 ◦C × 1 min, 72 ◦C × 1 min) 72 ◦C × 10 min, 4 ◦C
	sem	mpSEM-1<br>mpSEM-2	CTATTAATCTTTGGGTTAATGGAGAAC<br>TTCAGTTTCGACAGTTTTGTTGTCAT	300	95 ◦C × 5 min, 30 × (95 ◦C × 1 min, 55 ◦C × 1 min, 72 ◦C × 1 min) 72 ◦C × 10 min, 4 ◦C
	sen	mpSEN-1<br>mpSEN-2	ATGAGATTGTTCTACATAGCTGCAAT<br>AACTCTGCTCCCACTGAAC	680	
Exfoliative genes	eta	mpETA-1<br>mpETA-3	ACTGTAGGAGCTAGTGCATTTGT<br>TGGATACTTTTGTCTATCTTTTTCATCAAC	190	95 ◦C × 5 min, 30 × (95 ◦C × 1 min, 49 ◦C × 1 min, 72 ◦C × 1 min) 72 ◦C × 10 min, 4 ◦C
	etb	mpETB-1<br>mpETB-2	CAGATAAAGAGCTTTATACACACATTAC<br>AGTGAACTTATCTTTCTATTGAAAAACACTC	612	95 ◦C × 5 min, 30 × (95 ◦C × 1 min, 55 ◦C × 1 min, 72 ◦C × 1 min) 72 ◦C × 10 min, 4 ◦C
	siet	Siet1<br>Siet2	ATGGAAAATTTAGCGGCATCTGG<br>CCATTACTTTTCGCTTGTTGTGC	359	95 ◦C × 5 min, 35 × (95 ◦C × 45 s, 56 ◦C × 1 min, 72 ◦C × 1 min) 72 ◦C × 10 min, 4 ◦C
Hemolysins	α-haemolysin	HLA-1<br>HLA-2	CTGATTACTATCCAAGAAATTCGATTG<br>CTTTCCAGCCTACTTTTTTATCAGT	209	95 ◦C × 5 min, 30 × (95 ◦C × 1 min, 58 ◦C × 1 min, 72 ◦C × 1 min) 72 ◦C × 10 min, 4 ◦C
	β-haemolysin	HLB-1<br>HLB-2-2	GTGCACTTACTGACAATAGTGC<br>GTTGATGAGTAGCTACCTTCAGT	309	

	delta-hemolysin	HLD-1<br>HLD-2	AAGAATTTTTATCTTAATTAAGGAAGGAGTG<br>TTAGTGAATTTGTTCACTGTGTCGA	111	
	γ haemolysin A, B, and C	mpHLG-1<br>mpHLG-2	GTCATAGAGTCCATAATGCATTTAA<br>CACCAAATGTATAGCCTAAAGTG	535	95 ◦C × 5 min, 30 × (95 ◦C × 45 s, 55 ◦C × 45 s, 72 ◦C × 1 min) 72 ◦C × 10 min, 4 ◦C
	γ- Haemolysin variant	mpHLG2-1<br>mpHLG2-2	GACATAGAGTCCATAATGCATTTGT<br>ATAGTCATTAGGATTAGGTTTCACAAAG	390	
Leucocidins	PVL S and F	PVL-1<br>NPVL-2	ATCATTAGGTAAAATGTCTGGACATGATCCA<br>GCATCAASTGTATTGGATAGCAAAAGC		
	LukE-lukD	LUKDE-1<br>LUKDE-2	TGAAAAAGGTTCAAAGTTGATACGAG<br>TGTATTCGATAGCAAAAGCAGTGCA	269	95 ◦C × 5 min, 30 × (95 ◦C × 1 min, 58 ◦C × 1 min, 72 ◦C × 2 min) 72 ◦C × 10 min, 4 ◦C
	LukM	LUKM-1<br>LUKM-2	TGGATGTTACCTATGCAACCTAC<br>GTTCGTTTCCATATAATGAATCACTAC		
Adhesin factors	Clumping factors	clfA F<br>clfA R	GTAGGTACGTTAATCGGTT<br>CTCATCAGGTTGTTCAGG	1586	95 ◦C × 5 min, 30 × (95 ◦C × 30 s, 50 ◦C × 30 s, 72 ◦C × 1 min) 72 ◦C × 10 min, 4 ◦C
		clfB F<br>clfB R	TGCAAGATCAAACTGTTCCT<br>TCGGTCTGTAAATAAAGGTA	596	
	fibronectin binding protein fnb	FNBA-F<br>FNBA-R	GTGAAGTTTTAGAAGGTGGAAAGATTAG<br>GCTCTTGTAAGACCATTTTTCTTCAC	643	95 ◦C × 5 min, 30 × (95 ◦C × 30 s, 57 ◦C × 30 s, 72 ◦C × 1 min) 72 ◦C × 10 min, 4 ◦C
		FNBB-F<br>FNBB-R	GTAACAGCTAATGGTCGAATTGATACT<br>CAAGTTCGATAGGAGTACTATGTTC	524	
Fibrinogen binding protein		Fib1<br>Fib2	CTACAACTACAATTGCCGTCAACAG<br>GCTCTTGTAAGACCATTTTCTTCAC	404	95 ◦C × 5 min, 30 × (95 ◦C × 1 min, 57 ◦C × 1 min, 72 ◦C × 1 min) 72 ◦C × 10 min, 4 ◦C
Collagen binding protein		Cbp1<br>Cbp2	GTCAAGCAGTTATTAACACCAGAC<br>AATCAGTAATTGCACTTTGTCCACTG	423	95 ◦C × 5 min, 30 × (95 ◦C × 45 s, 62 ◦C × 45 s, 72 ◦C × 1 min) 72 ◦C × 10 min, 4 ◦C
Bone sialoprotein binding protein		bsp1<br>bsp2	AACTACATCTAGTACTCAACAACAG<br>ATGTGCTTGAATAACACCATCATCT	575	95 ◦C × 5 min, 30 × (95 ◦C × 45 s, 60 ◦C × 45 s, 72 ◦C × 1 min) 72 ◦C × 10 min, 4 ◦C
Laminin Binding Protein		Lamin1<br>Lamin2	ACGTGCAGCAGCTGACT<br>CAACAGCATTCTTCAGTACCTTC	302	95 ◦C × 5 min, 30 × (95 ◦C × 1 min, 55 ◦C × 1 min, 72 ◦C × 2 min) 72 ◦C × 10 min, 4 ◦C
Encoding elastin binding Protein		ebp1<br>ebp2	CATCCAGAACCAATCGAAGAC<br>CTTAACAGTTACATCATCATGTTTATCTTTG	186	95 ◦C × 5 min, 30 × (95 ◦C × 1 min, 55 ◦C × 1 min, 72 ◦C × 1 min) 72 ◦C × 10 min, 4 ◦C
Capsule proteins	*capsule type 5*	Cap5k1<br>Cap5k2	GTCAAAGATTATGTGATGCTACTGAG<br>ACTTCGAATATAAACTTGAATCAATGTTATACAG	361	95 ◦C × 5 min, 30 × (95 ◦C × 30 s, 50 ◦C × 30 s, 72 ◦C × 1 min) 72 ◦C × 10 min, 4 ◦C
	capsule type 8	Cap8k1<br>Cap8k2	GCCTTATGTTAGGTGATAAACC<br>GGAAAAACACTATCATAGCAGG	173	
